# Construction and evaluation of a combined model of NAA/STZ-induced type 2 diabetes with carotid balloon injury in SD rats

**DOI:** 10.3389/fendo.2025.1693820

**Published:** 2025-10-29

**Authors:** Yanmei Wang, Qian Zhou, Chengshan Li, Xiaojing Chenmou, Chan Zhu, Qiu Chen

**Affiliations:** ^1^ Clinical Teaching and Research Department, Ya’an Polytechnic College, Ya’an, Sichuan, China; ^2^ Department of Endocrinology, Hospital of Chengdu University of Traditional Chinese Medicine, Chengdu, Sichuan, China; ^3^ Internal Medicine Department II, Ya’an Polytechnic College Affiliated Hospital, Ya’an, Sichuan, China; ^4^ Department of Traditional Chinese Medicine, West China Second University Hospital, Sichuan University, Chengdu, Sichuan, China

**Keywords:** type 2 diabetes mellitus (T2DM), vascular balloon injury (VBI), neointimal hyperplasia, diabetic macroangiopathy, nicotinamide (NAA), streptozotocin (STZ)

## Abstract

**Purpose:**

Diabetic macroangiopathy is a leading cause of disability and mortality in type 2 diabetes mellitus (T2DM) patients, but existing animal models have limitations such as complex genetic manipulation or long induction cycles. This study constructed and evaluated a combined model of nicotinamide (NAA)/streptozotocin (STZ)-induced T2DM and carotid balloon injury in Sprague–Dawley (SD) rats.

**Methods:**

Sixty male SD rats were divided into four groups: control, VBI (only vascular balloon injury), T2DM (only NAA/STZ-induced T2DM), and T2DM+VBI (combined model) groups. T2DM was induced via NAA (110 mg/kg) followed by STZ (65 mg/kg); VBI was performed via a percutaneous transluminal coronary angioplasty (PTCA) balloon catheter. Over 6 weeks, metabolic indicators [intake, excretion, body weight, and random blood glucose (RBG)] were monitored; carotid tissues were collected at 2, 4, and 6 weeks post-VBI for HE staining and immunohistochemistry (α-SMA, F4/80, and MMP-9); and blood cell counts and plasma biochemistry were analyzed.

**Results:**

The T2DM induction success rate was 90% (no mortality); the postoperative mortality rate was 14.3% in the T2DM+VBI group (vs. 7% in the VBI group). The T2DM and T2DM+VBI groups presented typical diabetic symptoms and persistent hyperglycemia. Histologically, only the VBI and T2DM+VBI groups exhibited time-dependent neointimal proliferation, with T2DM+VBI exhibiting a larger neointimal area, a greater proliferation index, more severe luminal stenosis (2-week hyperplasia comparable to VBI 6 weeks) and increased F4/80-positive macrophages/MMP-9 (poor plaque stability). No major organ abnormalities (except T2DM hepatic steatosis) or intergroup blood parameter differences were found.

**Conclusion:**

This combined model has stable hyperglycemia, obvious neointimal hyperplasia, rapid modeling and a high success rate (optimal observation window: 2–4 weeks post-VBI). It recapitulates diabetic macroangiopathy pathology and is suitable for investigating therapeutic efficacy and related molecular/cellular mechanisms.

## 1 Introduction

Diabetic macroangiopathy is an atherosclerosis-related complication of long-term hyperglycemia, commonly affecting medium-sized arteries such as the coronary, cerebral basilar, carotid, renal, and lower limb arteries. In patients with type 2 diabetes, macroangiopathy is more prevalent and constitutes a major cause of disability and mortality (1–3). Hyperglycemia-induced oxidative stress and endothelial dysfunction promote the expression of adhesion molecules, chemokines, and other proinflammatory mediators, leading to extensive macrophage infiltration and the migration and proliferation of vascular smooth muscle cells. High glucose levels trigger glycosylation and the synthesis of oxidized low-density lipoprotein, further promoting foam cell formation in plaques, inducing smooth muscle apoptosis, and inhibiting collagen synthesis—all of which increase the risk of subsequent plaque rupture. Additionally, high glucose activates platelets and reduces the levels of endogenous platelet inhibitors, thereby promoting thrombosis (4, 5). Thus, diabetes is a well-recognized risk factor for atherosclerotic diseases, and cardiovascular events due to plaque rupture are significantly more common in diabetic patients, which is attributed to more extensive vascular involvement and diffuse atherosclerotic lesions (6).

The mechanisms underlying the progression from hyperglycemia to arterial inflammatory injury involve genetic, epigenetic, and cellular signaling pathways. Notably, oxidative stress and inflammation interact as key drivers (7). Reconstruction of this process requires a representative animal model. Rodents are widely used in biological and preclinical research because of their practical and economic advantages. However, mice lack cholesteryl ester transfer protein, leading to a distinct lipoprotein distribution compared with that of humans; this reduces circulating cholesteryl ester levels, protecting against atherosclerosis (8). Thus, generating atherosclerotic plaques in mice requires genetic manipulation, often involving crossbreeding with diabetic gene-deficient mice to model diabetic macroangiopathy—limiting large-scale application (9). A high-fat diet combined with streptozotocin (STZ) is commonly used to induce such models in rats, but this approach suffers from a long induction period, expensive feed, poor palatability, inadequate intimal hyperplasia, and failure to replicate atherosclerotic lesions optimally (10).

With the widespread implementation of percutaneous interventional therapy, clinical observations have shown that atherosclerotic plaque formation not only occurs naturally but also develops following arterial endotheliectomy, balloon angioplasty, and stent implantation (11). Subsequent animal studies demonstrated that balloon inflation and rotation can lead to complete denudation of vascular monolayer endothelial cells and that invasive neointima formation at the injury site follows a time-dependent pattern—this accelerates the spontaneous progression of atherosclerosis. Against this backdrop, the carotid balloon injury model was developed; rats are typically the preferred animal model, primarily because of their favorable size-related advantages (12). Vascular balloon injury (VBI) offers high reproducibility, controllable injury severity and length, simplicity, low postoperative mortality, and short modeling time, making it widely used in atherosclerosis research (13).

Nicotinamide (NAA) combined with STZ induction, first proposed by Masiello P et al. in 1998, was used to construct nonobese type 2 diabetes models in rats (14) and has since been extended to mice. It is widely used in studies of diabetes pathogenesis and complications and in preclinical drug screening (15, 16). Owing to the cytotoxicity of STZ to pancreatic β-cells and the global cytoprotective effect of NAA, this model leverages the contradictory effects of STZ on β-cells: early intervention with NAA can preserve a certain proportion of residual pancreatic β-cells, enabling them to release insulin under glucose stimulation (16, 17). While insulin resistance is lacking, it mimics pancreatic β-cell dysfunction in type 2 diabetes.

In this study, we constructed a combined model of NAA/STZ-induced type 2 diabetes with carotid balloon injury in Sprague–Dawley (SD) rats, monitored changes in metabolic indicators and the degree of arterial intimal thickening after modeling, and compared it with models of diabetes alone or balloon injury alone. A relatively comprehensive evaluation of this model has been performed.

## 2 Materials and methods

### 2.1 Material

Nicotinamide (NAA) and streptozotocin (STZ) were purchased from Sigma–Aldrich (St. Louis, MO, USA). Sodium citrate buffer (1 mol/L, pH 4.5, sterile) was obtained from Solarbio (Beijing, China). The primary antibodies used included rabbit anti-F4/80 (AR0279, Shenda Bio, China), rabbit anti-α-SMA (α-smooth muscle actin, 19245, CST, USA), and rabbit anti-MMP-9 (anti-matrix metalloproteinase-9, A11147, ABclonal, USA) antibodies. HRP-conjugated secondary antibodies were obtained from ZSGB-Bio (Beijing, China), and DAB dye was purchased from Dako (Denmark).

### 2.2 Animals

SD rats were purchased from Dashuo Experimental Animal Co., Ltd. (License No. SCXK 2020-30, Chengdu, China) and housed in an SPF environment with a 12 h light/dark cycle, 3–4 rats per cage, controlled temperature (23 ± 2 °C), and humidity (50–60%). All protocols were approved by the Experimental Animal Ethics Committee of the Affiliated Hospital of Chengdu University of Traditional Chinese Medicine (2021SDL-005).

### 2.3 Induction of type 2 diabetes in SD rats via the combination of NAA and STZ

SD rats were fasted overnight. After being weighed, they were intraperitoneally injected with 5% (w/v) NAA working solution at 110 mg/kg, followed by 1% (w/v) STZ working solution at 65 mg/kg 15 minutes later (14, 18). Random blood glucose (RBG) was measured 3 and 7 days post-modeling; values ≥ 16.7 mmol/L indicated successful induction (19, 20).

### 2.4 Establishment of a macroangiopathy model via carotid balloon injury

The rats were anesthetized with an intraperitoneal injection of 1% (w/v) pentobarbital sodium (40 mg/kg). After neck disinfection, a midline cervical incision was made, and the subcutaneous tissues and glands were bluntly dissected to expose the left sternocleidomastoid muscle. The common, external, and internal carotid arteries were identified, and silk thread was tied to temporarily occlude blood flow. A pressure pump was connected to a percutaneous transluminal coronary angioplasty (PTCA) balloon catheter to test airtightness, which was then evacuated to negative pressure for use. A “V”-shaped incision parallel to the vessel long axis was made 3 mm from the external carotid bifurcation. The balloon was advanced from the incision to the proximal common carotid artery, the common carotid occlusion was released, and the balloon was advanced 1–2 cm further. The pump was pressurized to 3–4 ATMs (with slight friction felt during rotation/retraction), and the balloon was withdrawn to the incision while rotating. This inflation–retraction cycle was repeated twice. After the third withdrawal, the balloon was deflated and removed, and then the external carotid proximal to the incision was ligated. The internal carotid occlusion was released to restore blood flow, confirmed by visible pulsation (12). The incision was sutured, and heparin (100 IU/kg) was injected intraperitoneally for anticoagulation. The rats were housed individually until recovery and then were grouped with other postoperative littermates. All balloon injuries were performed by the same surgeon.

### 2.5 Experimental design

#### 2.5.1 Grouping and treatment

**Figure 1:**
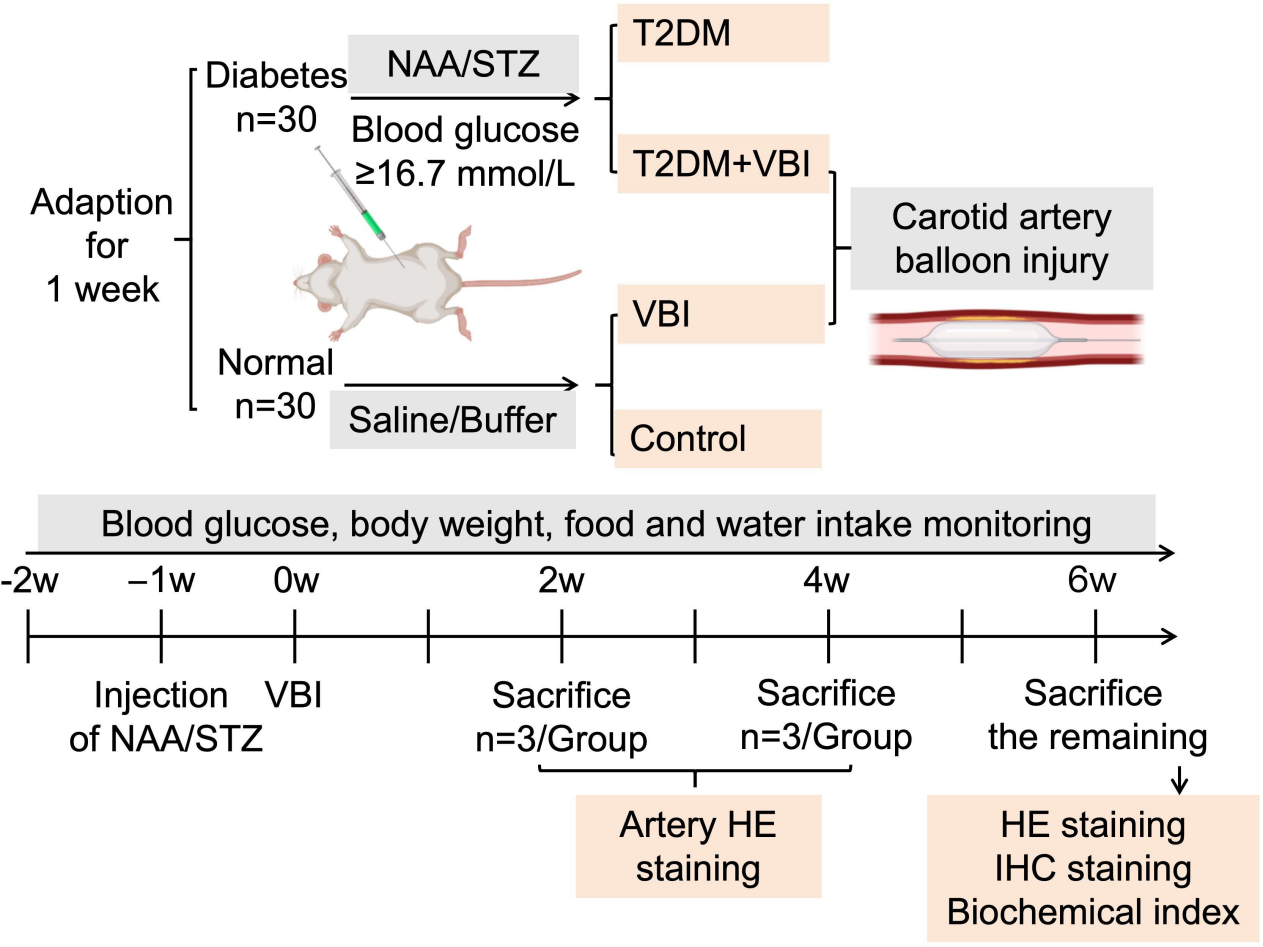
Experimental grouping and protocol. VBI, vascular balloon injury; T2DM, type 2 diabetes mellitus; NAA, nicotinamide; STZ, streptozotocin.

After 7 days of acclimation, 60 male SD rats (180–200 g) were randomly divided into normal and diabetic groups. The diabetes group received NAA followed by STZ; the normal group received 0.9% saline (1 mL/rat) and sodium citrate buffer (2 mL/rat) via intraperitoneal injection. After 1 week, diabetic rats (meeting the blood glucose criteria) were randomized to the T2DM+VBI (undergoing carotid balloon injury) or T2DM (diabetes control) groups; normal rats were randomized to the VBI (carotid balloon injury) or control (normal control) groups (**Figure 1**).

#### 2.5.2 Feeding protocol

The rats were fed standard rodent chow with ad libitum access to food and water. Daily water intake (mL/rat·d) was recorded. Cage bedding (corncob) was weighed before and after replacement to estimate daily excretion (g/rat·d). The remaining feed was weighed weekly, and fresh feed (1500 g) was provided to calculate the average daily food intake (g/rat·d). Five rats per group were weighed weekly, and tail vein RBG was measured.

At 2 and 4 weeks post-VBI, 3 rats per group were euthanized with 3% pentobarbital sodium, and the left common carotid artery was collected for histological staining. At 6 weeks post-VBI, the remaining rats were euthanized; abdominal aorta blood was collected for biochemical analysis, and the left common carotid artery and major organs were harvested for histological and immunohistochemical staining (**Figure 1**).

### 2.6 HE staining

Carotid arteries and organs were fixed in 4% (w/v) paraformaldehyde for ≥24 h, embedded in paraffin, and sectioned (4 μm). Hematoxylin and eosin (HE) staining was performed according to standard protocols. The sections were imaged via an automatic inverted fluorescence microscope (IX83, Olympus, Japan), and morphometric analysis was conducted with ImageJ (Fiji for Mac OS X, NIH, USA). Lumen, internal elastic lamina (IEL), and external elastic lamina (EEL) areas were measured by a blinded observer.

### 2.7 Immunohistochemical staining

Four-micrometer paraffin sections of injured arteries were deparaffinized, rehydrated, subjected to antigen retrieval, blocked, and then incubated overnight at 4 °C with the following primary antibodies: rabbit anti-F4/80 (1:100), anti-α-SMA (1:100), anti-MMP-9 (1:100), and anti-TNF-α (1:50). The sections were labeled with HRP-conjugated secondary antibodies and visualized with DAB. Images were captured via a microscope, and positive areas were quantified via ImageJ by a double-blinded investigator.

### 2.8 Statistical analysis

The data were analyzed via SPSS Statistics 26.0 (IBM, USA) and are expressed as the means ± standard deviations (SDs). Normality was tested via the Shapiro–Wilk test; nonparametric tests were used for nonnormal data. For comparisons between two groups, an unpaired t test was used; Welch’s correction was applied when variances were unequal. For comparisons among multiple independent groups, one-way ANOVA with Tukey’s *post hoc* test was employed; in cases of unequal variances, Brown–Forsythe/Welch ANOVA with Dunnett’s T3 test was used instead. To compare the mean differences between groups with multifactorial interactions, two-way ANOVA was performed; if the sphericity assumption was violated, Geisser–Greenhouse correction was adopted, and Tukey’s test was used for pairwise comparisons between groups. A value of P < 0.05 was considered statistically significant.

## 3 Results

### 3.1 General conditions and metabolic indicators

The rats in the control group generally maintained good health: they appeared lively and robust, with clean, glossy fur, and no spontaneous deaths occurred during the feeding period. In the VBI group, the rats exhibited mild lethargy on the second day after carotid balloon injury surgery but were not different from normal rats for the remainder of the observation period. Only one rat in this group died from anesthesia prior to model establishment, resulting in a mortality rate of 7%. For the rats subjected to diabetes induction (via STZ combined with NAA injection), no deaths occurred within one week post-injection. Among the 30 rats, 27 met the predefined blood glucose criteria, yielding a diabetes model success rate of 90%. Subsequently, 14 adult diabetic rats were randomly selected and subjected to carotid balloon injury surgery (assigned to the T2DM+VBI group). One rat died intraoperatively, and another died during postoperative resuscitation, leading to a mortality rate of 14.3%. The remaining 13 diabetic rats were allocated to the T2DM group. No deaths occurred in either the T2DM+VBI group or the T2DM group during the subsequent feeding period.

**Figure 2:**
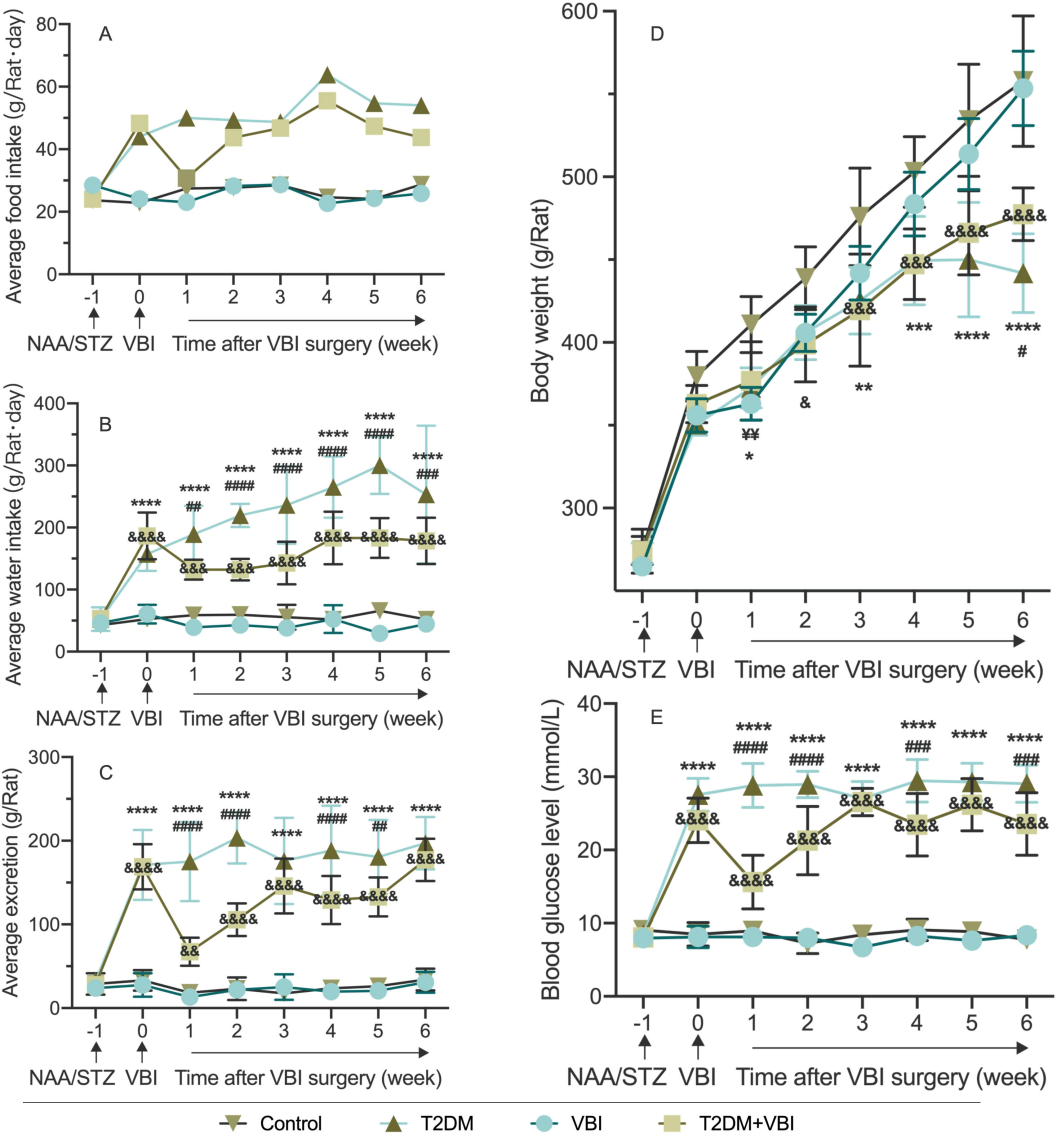
Comparison of average food intake. **(A)**, Water intake **(B)**, excretion **(C)**, body weight **(D)**, and random blood glucose **(E)** in rats after modeling. VBI, vascular balloon injury; T2DM, type 2 diabetes mellitus; NAA, nicotinamide; STZ, streptozotocin. Two-way ANOVA was performed for statistical comparison, and Geisser–Greenhouse correction was adopted if the sphericity assumption was violated: *: Comparison between the T2DM group and the control group (*P < 0.05; **P < 0.01; ***P < 0.001; ****P < 0.0001). & Comparison between the T2DM+VBI group and the control group (&P < 0.05; &&P < 0.01; &&&P < 0.001; &&&&P < 0.0001); ¥: Comparison between the VBI group and the control group (¥¥P < 0.01); #: Comparison between the T2DM+VBI group and the T2DM group (#P < 0.05; ##P < 0.01; ###P < 0.001; ####P < 0.0001).

As shown in **Figure 2A** and **Supplementary Table 1**, the control and VBI groups had similar food intakes (~25 g/rat·d). The T2DM and T2DM+VBI groups presented significantly increased intake postmodeling: T2DM remained at ~50 g/rat·d, whereas T2DM+VBI intake decreased slightly 1 week post-injury and then approached T2DM levels. Since food intake was recorded only once per week, this measurement could reflect only the overall trend and did not allow for the calculation of significant differences.

Water intake was similar in the VBI and control groups (~50 mL/rat·d, not significant; **Figure 2B**). The T2DM and T2DM+VBI groups presented significantly increased water intake postmodeling (P < 0.001 vs. Control). T2DM intake remained >150 mL/rat·d, whereas T2DM+VBI intake decreased postinjury and then rose slowly but remained higher than Control and lower than T2DM intake (P < 0.01 vs. Control and P < 0.05 vs. T2DM at all post-VBI time points, **Figure 2B** and **Supplementary Table 2**).

The VBI and control cages were clean with formed feces; the T2DM and T2DM+VBI cages were dirty with loose stools. The weight increase of the cushion material determined by weighing the cage boxes was similar between the VBI and control groups (**Figure 2C**). The excretion of the T2DM and T2DM+VBI groups increased significantly post-modeling (P < 0.01 vs. Control). T2DM+VBI excretion decreased postinjury and then approached T2DM levels, with significant differences at 1, 2, 4, and 5 weeks post-VBI (P < 0.05, **Supplementary Table 3**).

Body weight increased in all groups over the observation period (**Figure 2D**). Specifically, the body weight of the VBI group was significantly lower than that of the control group only at 1 week post-injury (P < 0.01). The body weight of the T2DM group was lower than that of the control group at week 1 and weeks 3–6 (P < 0.05). In the T2DM+VBI group, the body weight was lower than that in the control group starting at 2 weeks post-VBI (P < 0.05); this group showed a consistent trend of change with the T2DM group, with statistical significance observed only at week 6 (P < 0.05, **Supplementary Table 4**).

The random blood glucose levels of the control and VBI groups were essentially identical, with both remaining stable at baseline levels throughout the entire experimental period; no significant differences were observed between these two groups (**Figure 2E**). Following modeling with STZ combined with NAA, the RBG levels of the rats in the T2DM and T2DM+VBI groups increased significantly. Compared with the control group, both groups presented statistically significant differences in blood glucose at all time points after diabetes induction (P < 0.001). For the T2DM group, blood glucose levels remained above 25 mmol/L and exhibited no significant fluctuations over time. After balloon injury surgery, the RGB level of the T2DM+VBI group decreased to 15.62 ± 3.66 mmol/L; although it gradually increased again, it remained slightly lower than that of the T2DM group (P < 0.05 at 1–2, 4, and 6 weeks post-VBI, **Supplementary Table 5**).

### 3.2 Comparison of carotid artery histomorphology

**Figure 3:**
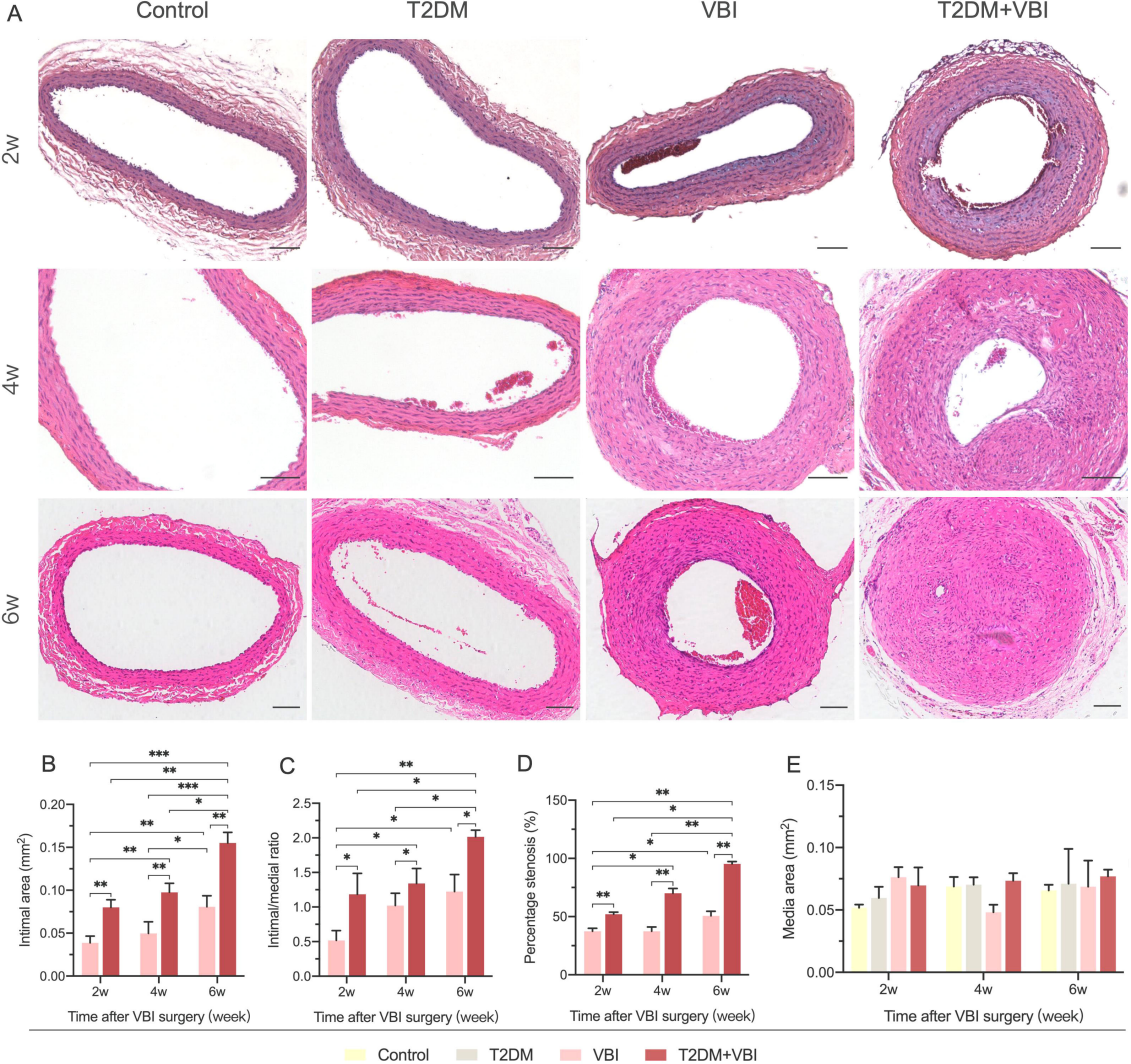
Comparison of carotid artery histomorphology (HE Staining) after balloon injury. **(A)** Representative HE-stained images of the carotid artery at 2, 4, and 6 weeks post-balloon injury in each group. **(B)** Comparison of the neointimal area at different time points after surgery among the groups. **(C)** Comparison of the arterial proliferation index at different time points after surgery among the groups. **(D)** Comparison of the percentage of carotid artery stenosis at different time points after surgery among the groups. **(E)** Comparison of the arterial media area at different time points after surgery among the groups. VBI, vascular balloon injury; T2DM, type 2 diabetes mellitus. Scale bar = 100 μm. Two-way ANOVA was performed for statistical comparison, and Geisser–Greenhouse correction was adopted if the sphericity assumption was violated, *P < 0.05; **P < 0.01; ***P < 0.001.

At 2, 4, and 6 weeks after vascular injury, samples were harvested from the left common carotid artery of the rats in each group for histological examination. Owing to varying degrees of vascular lumen compression during paraffin embedding, which led to a reduced lumen deformation area, the measured lumen area was only used for calculating the intimal proliferation area and was not subjected to statistical analysis. In addition, no obvious intimal proliferation was observed in sections from rats in the normal control group or diabetes group; the monolayer vascular endothelium was closely attached to the internal elastic membrane, making it impossible to accurately distinguish the boundary between the two. Therefore, the intimal area and its related calculations were only performed for the VBI group and T2DM+VBI group. Two weeks after balloon injury, the carotid intima began to proliferate to varying degrees. In the presence of diabetes, the proliferation area increased with increasing injury time (**Figure 3A**). When the two groups were compared, intimal proliferation was more pronounced in diabetic rats after balloon injury; by 6 weeks post-injury, the vascular intima in most cases had thickened to a degree that nearly occluded the lumen and disrupted blood flow. Quantitative analysis revealed that at 2, 4, and 6 weeks post-injury, the neointimal area (IEL area–lumen area) in the T2DM+VBI group was significantly larger than that in the VBI group (P<0.01, **Figure 3B**). Similarly, statistical analysis of the proliferation index (neointimal area/media area) and stenosis percentage [1 – (lumen area/IEL area)] (21, 22) revealed that both parameters were significantly greater in the T2DM+VBI group than in the VBI group, with statistically significant differences at each time point (P<0.01, **Figures 3C, D**). Notably, at 2 weeks post-injury, the neointimal area, proliferation index, and stenosis percentage in the T2DM+VBI group were comparable to those in the VBI group at 6 weeks post-injury, with no statistically significant differences (2w T2DM+VBI group vs. 6w VBI group: neointimal area=0.080 ± 0.009 mm² vs. 0.081 ± 0.013 mm², P = 0.9983; proliferation index=1.19 ± 0.30 vs. 1.22 ± 0.25, P = 0.9247; stenosis percentage=52.10 ± 1.68% vs. 50.63 ± 3.93%, P = 0.9995). In addition, there was no statistically significant difference in the arterial media area (EEL area – IEL area) among the different sampling time points (**Figure 3E**).

### 3.3 Immunohistochemical analysis of neointima

**Figure 4:**
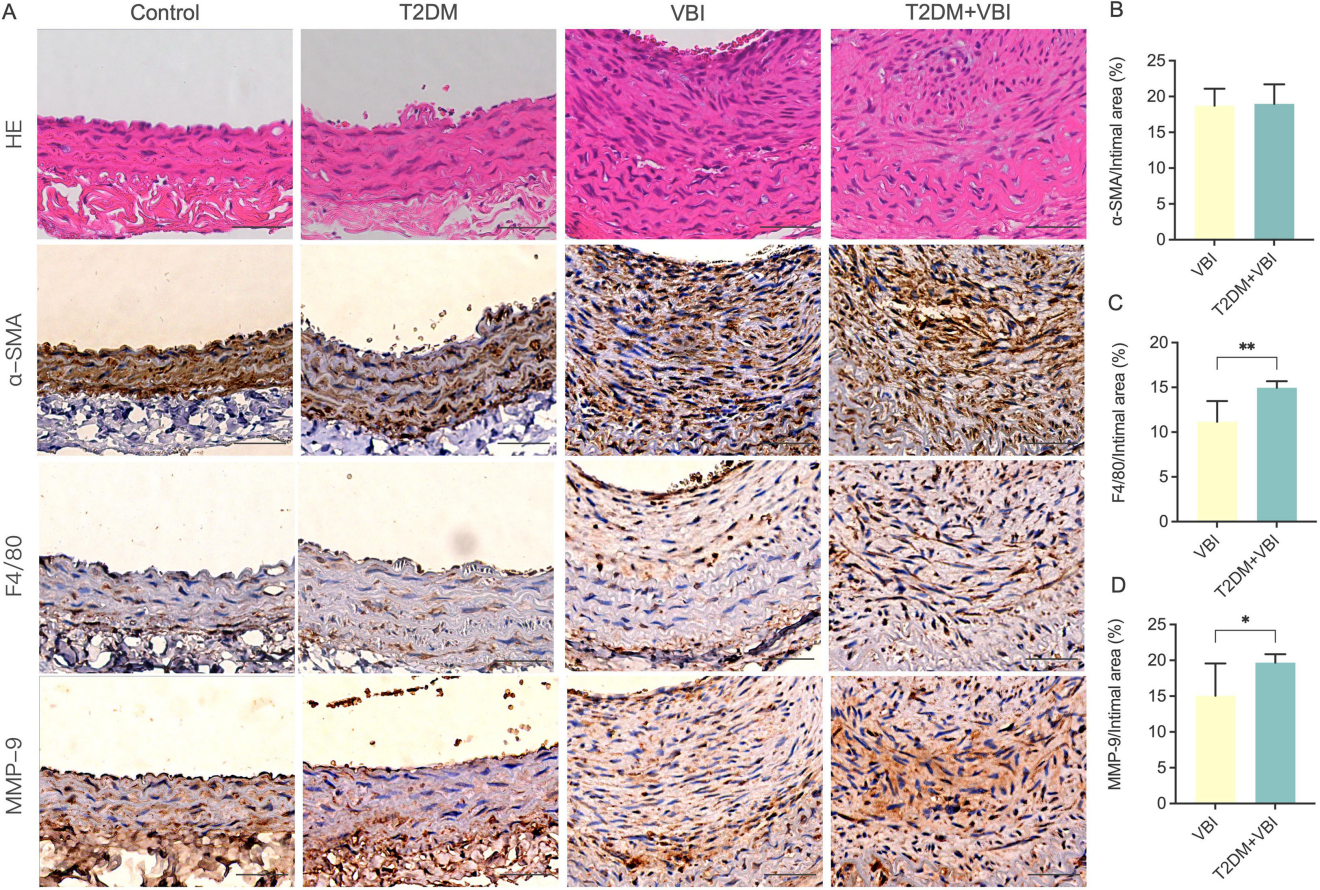
Comparison of carotid artery histopathological and immunohistochemical staining at 6 weeks post-balloon injury. **(A)** Representative images of immunohistochemical staining of the carotid artery in each group. **(B)** Ratio of the α-SMA-positive area to the neointimal area. **(C)** Ratio of the F4/80-positive area to the neointimal area. **(D)** Ratio of the MMP-9-positive area to the neointimal area. VBI, vascular balloon injury; T2DM, type 2 diabetes mellitus; α-SMA, α-smooth muscle actin; F4/80, macrophage marker; MMP, matrix metalloproteinases. Scale bar = 50 μm. Unpaired t tests were used for statistical comparisons, and Welch’s correction was applied when variances were unequal, *P < 0.05, **P < 0.01.

To further analyze and compare the compositional and structural changes in proliferating vascular endothelial cells, immunohistochemical staining was performed for the classical vascular smooth muscle cell molecular marker α-SMA and the macrophage-specific marker F4/80 in carotid artery tissues obtained from the rats in each group at 6 weeks post-injury. As shown in **Figure 4A**, in the control and T2DM groups, the arterial wall was smooth, the internal elastic lamina remained intact, and endothelial cells uniformly covered the IEL surface. The cells in the arterial media were arranged neatly, with abundant α-SMA expression detected between the internal and external elastic laminae. F4/80-positive cells were only scattered in an extremely sparse distribution. In the VBI group, despite significant thickening of the vascular intima, the spindle-shaped cells within the intima were relatively uniformly arranged and clustered on the luminal side. In contrast, the T2DM+VBI group presented unclear cellular structure and markedly disorganized arrangement in the thickened intima. Notably, both the VBI and T2DM+VBI groups presented prominent α-SMA expression in the proliferative intimal region, indicating that neointimal formation is driven primarily by the migration and proliferation of vascular smooth muscle cells. Quantitative analysis revealed no statistically significant difference in the proportion of the α-SMA-positive stained area within the neointima between the two groups (VBI group vs. T2DM+VBI group: 18.71 ± 2.376% vs. 18.98 ± 2.710%, P = 0.7577; **Figures 4A, B**). In F4/80-stained sections, the number of F4/80-positive cells in the proliferative intimal region was significantly greater in the T2DM+VBI group than in the VBI group (VBI group vs. T2DM+VBI group: 11.17 ± 2.297% vs. 14.97 ± 0.7204%, P = 0.0125), suggesting enhanced macrophage infiltration and more severe inflammatory responses (**Figures 4A, C**). Additionally, when the samples were stained with antibodies against matrix metalloproteinase-9 (MMP-9), extensive positive staining was also observed in the T2DM+VBI group. Compared with that in the VBI group, the proportion of MMP-9-positive stained area in the neointima in the VBI group was significantly different (VBI group vs. T2DM+VBI group: 15.07 ± 4.491% vs. 19.68 ± 1.183%, P = 0.0049; **Figures 4A, D**).

### 3.4 HE Staining of major organs

**Figure 5:**
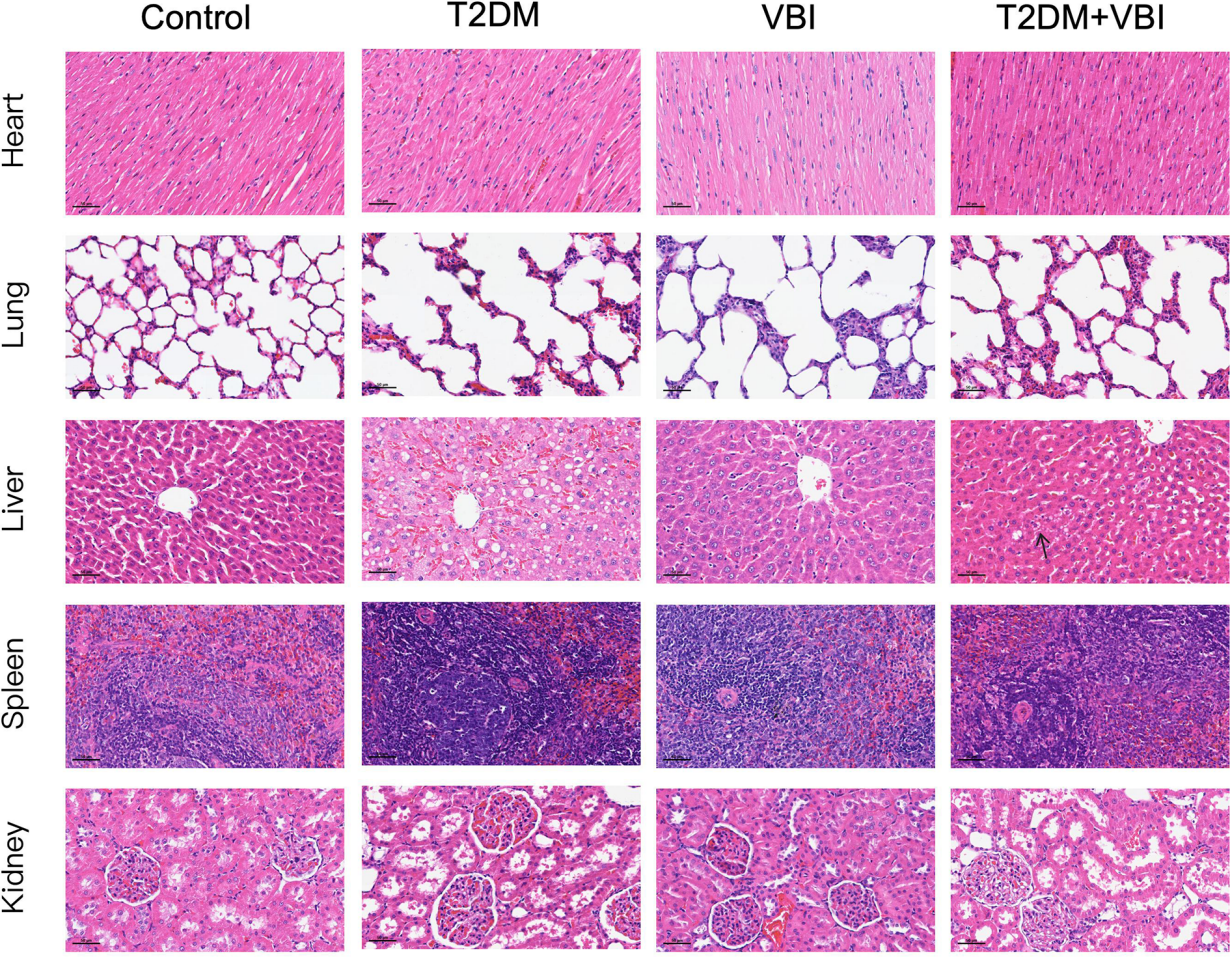
HE-stained sections of major organs from each group at 6 weeks post-VBI. VBI, vascular balloon injury; T2DM, type 2 diabetes mellitus. Scale bar = 50 μm.

As shown in **Figure 5**, no significant histomorphological abnormalities were observed in hematoxylin–eosin (HE)-stained sections of the heart, lungs, spleen, or kidneys across all treatment groups; specifically, no pathological changes (e.g., cell degeneration, necrosis, or inflammatory cell infiltration) were detected. In HE-stained liver sections from the T2DM group, marked hepatocellular swelling with steatosis was observed, and some hepatocytes exhibited reticular fragmentation or even ballooning degeneration. Concurrently, numerous vacuoles (resulting from lipid droplet dissolution) were noted. In contrast, the T2DM+VBI group exhibited relatively mild hepatocellular injury, with only scattered, loosely arranged, and edematous hepatocytes (indicated by arrows); the overall cellular arrangement was comparable to that of the VBI group and the control group.

### 3.5 Whole blood cell and plasma biochemical analyses

**Figure 6:**
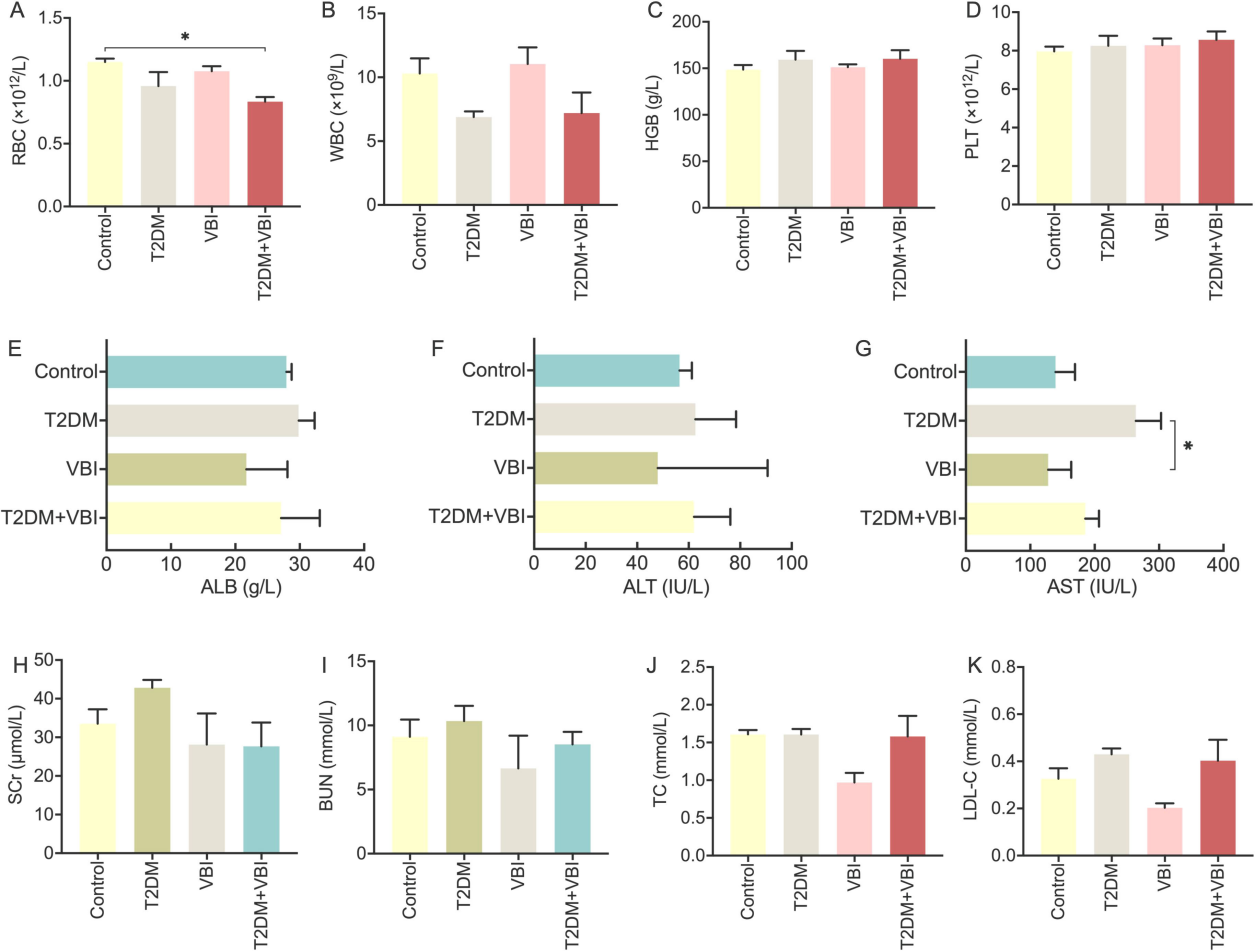
Analysis and comparison of whole blood cell and plasma biochemical parameters in diabetic rats at 6 weeks post-VBI. **(A)** RBC: red blood cell count analysis; **(B)** WBC: total white blood cell count analysis; **(C)** HGB: hemoglobin quantitative analysis; **(D)** PLT: platelet count analysis; **(E)** ALB: plasma albumin measurement; **(F)** ALT: alanine aminotransferase assay; **(G)** AST: aspartate aminotransferase assay; **(H)** SCr: plasma creatinine measurement; **(I)** BUN: plasma urea nitrogen determination; **(J)** TC: plasma total cholesterol concentration; **(K)** LDL-C: plasma low-density lipoprotein cholesterol measurement. VBI, vascular balloon injury; T2DM, type 2 diabetes mellitus. One-way ANOVA with Tukey’s *post hoc* test and Brown–Forsythe/Welch ANOVA with Dunnett’s T3 test were used for statistical comparison, *P < 0.05.

Analysis of whole blood cell parameters in the rats across all the groups revealed the following: after STZ combined with NAA, the red blood cell (RBC) and white blood cell (WBC) counts in the T2DM and T2DM+VBI groups decreased to varying extents compared with those in the control group and the VBI group; however, these differences did not reach statistical significance. No significant intergroup differences were observed in hemoglobin (HGB) or platelet (PLT) levels (**Figures 6A–D**).

Liver and kidney function analyses: Rats in the T2DM group presented significant increases in plasma aspartate transaminase (AST) levels, along with slight increases in plasma creatinine (Cr) and urea nitrogen (UN) levels. Nevertheless, these changes were not statistically significant compared with those of the other groups. Additionally, liver and kidney function parameters in the T2DM +VBI group were comparable to those in the control group, with no significant differences detected (**Figures 6E–I**).

Lipid metabolism: At 6 weeks post-modeling, the VBI group presented slight decreases in plasma total cholesterol (TC) and low-density lipoprotein (LDL) levels, although these reductions were not statistically significant. Moreover, the plasma lipid and lipoprotein levels in the T2DM and T2DM+VBI groups were comparable to those in the control group, with no significant intergroup differences observed (**Figures 6J, K**).

## 4 Discussion

Previous studies have applied arterial balloon injury intervention to STZ-induced diabetic animal models to investigate its therapeutic efficacy and underlying mechanisms; however, a comprehensive evaluation of this combined model is lacking (23–25). In the present study, we successfully established a combined model of NAA/STZ-induced type 2 diabetes mellitus and carotid balloon injury (T2DM+VBI model) in SD rats. On the basis of this model, we conducted a relatively comprehensive investigation to compare time-dependent arterial intimal thickening and postinjury changes in metabolic indicators between the T2DM+VBI group and two control groups: the T2DM group (SD rats with NAA/STZ-induced T2DM but no VBI) and the VBI group (SD rats with VBI but no T2DM). This work lays a foundation for the subsequent promotion and application of this combined model in studies exploring drug efficacy and potential mechanisms.

For diabetes induction, we used a combination of NAA at 110 mg/kg and STZ at 65 mg/kg. Although the initially proposed doses of these two agents are 230 mg/kg and 65 mg/kg, respectively (14), the NAA dose was reduced in practice to weaken its protective effect (NAA exhibits a 100% protective effect against STZ-induced pancreatic β-cell damage at adequate doses); this adjustment was intended to improve the diabetes induction rate. Additionally, the key to the combined use of NAA and STZ lies in standardizing the dosage, administration route, and timing across all experimental animals, which ensures the comparability of metabolic data between groups.

Streptozotocin (STZ) is widely utilized in chemically induced diabetes models. Owing to its structural similarity to glucose, STZ acts as a glucose analog and is selectively taken up by pancreatic β-cells via glucose transporter-2 (GLUT2), a membrane protein abundantly expressed on the β-cell surface. Upon entering β-cells, the nitrosoamide moiety of STZ exerts genotoxic and cytotoxic effects by attacking genomic DNA and inducing alkylation. This STZ-mediated DNA damage triggers excessive activation of poly-ADP-ribose-polymerase-1 (PARP-1), an enzyme that utilizes nicotinamide adenine dinucleotide (NAD+) as a substrate to catalyze poly-ADP-ribosylation. Sustained PARP-1 overactivation depletes intracellular NAD+ and adenosine triphosphate (ATP), ultimately resulting in β-cell necrosis (16). The sirtuin (SIRT) family of enzymes also relies on NAD+ as a cosubstrate to catalyze the deacylation of lysine residues on target proteins, generating NAA and 2-O-acetyl-ADP-ribose as byproducts (26). Among SIRTs, SIRT1 plays a pivotal role in cellular defense against oxidative stress and in modulating insulin sensitivity and β-cell function in the context of the pathophysiological network of nonalcoholic fatty liver disease and T2DM (17). Notably, both the PARP-1 and SIRT enzymes share NAD+ as a critical substrate and produce endogenous NAA as a common product; thus, their activities can be inhibited by exogenous NAA (17, 18, 26). Leveraging this NAA-induced suppression, the combination of STZ and NAA achieves two key objectives for T2DM modeling: downregulating PARP-1 activation to preserve residual β-cells and inhibiting SIRT1 to mimic insulin resistance. As a model of nonobese T2DM, the STZ + NAA system has distinct advantages for diabetes research: it maintains stable moderate hyperglycemia without requiring exogenous insulin for animal survival, retains partial glucose-stimulated insulin secretion capacity, and responds to sulfonylureas. These features make it highly suitable for evaluating the biochemical and pharmacological effects of potential antidiabetic agents in a model that mirrors key characteristics of human T2DM (16).

For the evaluation of diabetes model success, we adopted an RBG threshold of ≥ 16.7 mmol/L as the criterion, which was determined on the basis of two considerations. First, rats exhibited reduced food intake postballoon injury, and fasting blood glucose monitoring (which requires food restriction) further increased animal mortality. Thus, random blood glucose monitoring was used, and consistency was maintained throughout the feeding period. Second, blood glucose measurement in rats is frequently accompanied by stress, and the baseline RBG of normal rats fluctuates approximately 9 mmol/L, approaching the clinical diagnostic threshold of 11.1 mmol/L for random blood glucose in patients with diabetes mellitus. Furthermore, our preliminary experiments (data not shown) revealed that rats with RBG levels between 11.1 and 16.7 mmol/L tended to spontaneously revert to normoglycemia during the diabetic feeding period. These factors collectively justified the use of an RBG level of 16.7 mmol/L as a criterion for successful diabetes induction. On the basis of the aforementioned induction protocol and criteria, this study achieved a high diabetes induction rate (90%) and a low mortality rate (0%) during the diabetes modeling phase.

For carotid balloon injury modeling, we employed a 1.5 × 12 mm PTCA balloon dilation catheter. Compared with traditional Fogarty balloon thrombectomy catheters, PTCA balloons are more cost-effective and readily accessible to our research team. Historically, the rat carotid artery balloon injury model was primarily established via Fogarty balloon embolectomy catheters, as early PTCA balloons were generally too large to be applied in small animal models (12, 27). Notably, the two types of balloons differ significantly in their design and functional characteristics. The Fogarty balloon exhibits high compliance, expanding both radially (along the vessel diameter) and longitudinally (along the vessel length) upon inflation, which makes it suitable for surgical thrombectomy (27, 28). In contrast, PTCA balloons—originally developed for coronary artery interventions—possess slight compliance and can maintain their inflated size even as pressure increases, and when paired with a pressure pump, they enable precise and controlled dilation (27, 29). Considering that previous studies have confirmed that Fogarty balloons and PTCA balloons induce comparable levels of arterial wall damage (27, 30), PTCA balloons are now feasible for carotid angioplasty in rats owing to advancements in catheter technology—particularly in size, specifications, and materials (29). Even among personnel with extensive experience in arterial balloon injury procedures, the mortality rate remained higher in the diabetic group than in the nondiabetic group (14.3% vs. 7%). This difference may be attributed to increased platelet aggregation and thrombotic risk, which are driven by elevated oxidative stress levels and activated proinflammatory mediator levels in diabetic animals (31). Given the above considerations, it is necessary to appropriately increase the intragroup sample size during actual experiments, with adjustments on the basis of pre-experimental data regarding diabetes induction rates and animal mortality.

Throughout the entire feeding period, we continuously monitored and systematically recorded changes in diabetes-related metabolic indicators in the rats across all the groups. Considering that urine monitoring via metabolic cages may disrupt rats’ daily activities and is not sustainable, we adopted a cage weighing method to estimate excretion. Although this method yields data with lower precision than metabolic cages do, it offers a clear advantage over qualitative observation by enabling quantitative estimation of excretion (32). Observations revealed that rats in the nondiabetic groups—regardless of whether they received carotid balloon injury—exhibited no significant fluctuations in food intake, water intake, excretion, or blood glucose levels, with consistent trends in body weight gain. These findings indicate that carotid balloon injury has no significant effect on the daily activities or basic metabolic homeostasis of nondiabetic rats. In contrast, rats with NAA/STZ-induced T2DM presented typical diabetic symptoms (polydipsia, polyphagia, and polyuria) within one week of modeling, accompanied by a significant elevation in blood glucose levels. For the T2DM group, the aforementioned indicators (food intake, water intake, excretion, and blood glucose) remained consistently high, with only occasional minor fluctuations. In the T2DM+VBI group, the rats exhibited a one-week decrease in food intake, water intake, and excretion after balloon injury surgery; although their blood glucose levels also decreased significantly during this period, they remained significantly higher than those in the control group. These indicators (intake, excretion, and blood glucose) subsequently gradually increased; however, while they did not reach the levels observed in the T2DM group, they were still significantly higher than those in the control group. Notably, starting from the second week post-VBI, the blood glucose levels of the T2DM+VBI group remained above the threshold for successful diabetes induction. In terms of body weight, the T2DM+VBI group and T2DM-only group presented similar trends: both groups presented significantly lower body weights than the control group did, with this significant difference first observed around the third week after diabetes induction (corresponding to the second week after balloon injury). These results validated the efficacy of the NAA/STZ-induced T2DM model: in addition to displaying typical diabetic symptoms, the diabetic rats maintained a sustained increase in blood glucose levels. After balloon injury, the metabolic indicators of diabetic rats were partially affected, but their blood glucose levels still met the criteria for a stable diabetic model, and they continued to exhibit prominent diabetic symptoms. Additionally, because metabolic indicators in the T2DM+VBI group remained consistently lower than those in the T2DM-only group postballoon injury, we hypothesize that this may be related to the transient reduction in food intake caused by surgical stress. This reduction may allow pancreatic islet cells adequate rest for partial functional recovery, thereby moderately mitigating metabolic abnormalities (33).

To assess the impact of diabetic model on intimal hyperplasia severity at the balloon injury site, we harvested the common carotid arteries of the rats in each group at 2, 4, and 6 weeks after vascular injury for histological analysis. The results revealed no proliferative lesions in carotid artery sections from the rats in the normal control group or the T2DM group. The arterial walls were smooth, the internal elastic membrane was intact, and no luminal stenosis was observed. In rats subjected to carotid balloon injury, varying degrees of proliferative lesions were evident 2 weeks post-surgery, and the proliferative area gradually expanded as the postinjury period increased. At each test time point, the degree of carotid intimal proliferation and luminal stenosis was significantly greater in diabetic rats than in those subjected to balloon injury only. By 6 weeks after balloon injury, the vascular intima in most diabetic rats had thickened to nearly occlude the lumen and disrupt blood flow. This result confirmed that the diabetic milieu can accelerate the development of intimal proliferative lesions after endothelial injury. Notably, the carotid intimal proliferation data in the T2DM+VBI group at 2 weeks post-injury were comparable to those in the VBI group at 6 weeks, which also indicates that carotid artery injury in the context of diabetes can further shorten the modeling time and that 2–4 weeks after balloon injury is the appropriate modeling period.

For subsequent immunohistochemical analyses, we stained vascular tissues harvested at 6 weeks post-injury for the vascular smooth muscle cell marker α-SMA and the macrophage marker F4/80. The results confirmed that, regardless of the presence or absence of diabetes, the primary cellular components of the proliferative intima were macrophages and vascular smooth muscle cells. Notably, the proliferation and infiltration of these two cell types were more prominent in diabetic rats than in control rats. In addition, α-SMA staining in the VBI group revealed aggregation and bundling of spindle-shaped cells on the luminal side of the endothelium, a finding considered indicative of fibrous cap formation and a hallmark of stable plaques. In contrast, the T2DM+VBI group presented unclear cellular structure and significantly disorganized arrangement within the thickened intima. For further investigation, we stained the neointima with MMP-9, a representative marker of plaque rupture and remodeling, and observed a larger area of positive expression in the T2DM+VBI group, indicating that in the diabetic milieu, the proliferative intima undergoes more severe inflammatory damage, and the lesions exhibit relatively poor stability. Notably, the homolog of F4/80 in human tissues serves as a highly specific marker for eosinophils and is undetectable in monocytes or macrophages (34). Thus, when researchers aim at cross-species comparisons, CD68 is a more appropriate choice, as this marker is consistently expressed across humans, mice, and rats (35, 36). Although CD68 is not as specific as F4/80 (34, 37), it still provides a better bridge for translational relevance between rodent and human studies. The failure to combine the detection of CD68 and F4/80 during the initial experimental design is a limitation of our study.

Six weeks after balloon injury, histological examinations were performed on the major organs of the rats in each group. In HE-stained liver sections, hepatocytes in the T2DM group presented significant fatty degeneration and ballooning changes, whereas only scattered, loosely arranged, and edematous cells were observed in the T2DM+VBI group. No obvious abnormalities were found in other organs across all groups. Additionally, comparative analyses of whole blood cell count and plasma biochemical parameters revealed no statistically significant differences between the groups. Even in the absence of significant differences in hematological, biochemical, and histological assessments, these results hold substantial value for validating the translational relevance of the combined model. Specifically, they confirm that the model avoids inducing acute, severe organ injury—including overt hepatic/renal dysfunction and severe dyslipidemia. This is critical because such severe pathological states are not characteristic of early-stage T2DM-associated complications in humans; instead, they introduce confounding variables that overwhelm the specific metabolic-related effects of T2DM itself on vascular injury. Notably, nonsignificant differences do not equate to “no biological effect”: mildly elevated ALT/AST levels paired with histological evidence of mild steatosis would mirror early-stage T2DM-related liver dysfunction, and nonsignificant BUN/SCr differences do not exclude early DN. Integrating advanced functional assessments (e.g., GFR) and histological evaluations (e.g., PAS staining for kidneys, oil red O staining for liver) would enhance the model’s ability to recapitulate the full spectrum of T2DM-associated pathologies (17, 18). On the basis of the current dataset, the combined model successfully maintains organ function within the range of subclinical dysfunction during the early stages of T2DM and vascular injury.

## 5 Conclusion

In this study, we successfully established an SD rat model of NAA/STZ-induced type 2 diabetes combined with carotid artery balloon injury and proposed that 2–4 weeks after balloon injury constitutes a relatively appropriate time window for model establishment. Consistent with the metabolic profiles of rats with simple diabetes, this model displays persistently elevated blood glucose levels and distinct diabetic symptoms from 2 weeks post-injury onward. Compared with those in rats subjected to balloon injury alone, the injured intima in the diabetic environment shows accelerated proliferation, with more severe intimal hyperplasia and lumen stenosis, more prominent inflammatory cell infiltration and proliferation, and poor stability of the proliferative intima. Overall, this model offers advantages such as significant intimal hyperplasia, short modeling duration, stable blood glucose levels, high modeling success rate, and low cost. It can be applied to future research on potential drug effects and their mechanisms.

## Data Availability

The original contributions presented in the study are included in the article/**Supplementary Material**. Further inquiries can be directed to the corresponding author.
