# Supplementary material for: Construction and evaluation of a combined model of NAA/STZ-induced type 2 diabetes with carotid balloon injury in SD rats
Source: Front Endocrinol (Lausanne). 2025 Oct 29;16:1693820. doi: 10.3389/fendo.2025.1693820 (PMC12605494; doi:10.3389/fendo.2025.1693820)
Supplement: Supplementary file 1 [file Table1.docx]

Supplementary Material

**Supplementary Table 1** Comparison of Food Intake (g/rat·day).

| Time Point | Control group | T2DM group | VBI group | T2DM+VBI group |
| --- | --- | --- | --- | --- |
| -1 week | 25.33 | 25.33 | 23 | 30.75 |
| 0 week | 44 | 44 | 28.25 | 43.66 |
| 1 week | 50 | 50 | 28.66 | 46.75 |
| 2 week | 49.33 | 49.33 | 22.66 | 55.5 |
| 3 week | 48.66 | 48.66 | 24.33 | 47.375 |
| 4 week | 63.83 | 63.83 | 25.83 | 43.766 |
| 5 week | 54.73 | 54.73 | 23 | 30.75 |
| 6 week | 54 | 54 | 28.25 | 43.66 |

**Supplementary Table 2** Comparison of Water Intake (mL/rat·day).

| Time Point | Control group | T2DM group | VBI group | T2DM+VBI group |
| --- | --- | --- | --- | --- |
| -1 week | 42.86±8.91 | 52.38±19.22 | 46.07±13.19 | 53.33±9.81 |
| 0 week | 52.38±4.18 | 157.14±26.86^a^ | 60.48±14.96^b^ | 186.43±37.75^a,c^ |
| 1 week | 58.93±12.4 | 189.29±45.89^a^ | 39.05±7.87^b^ | 132.14±15.91^a,b,c^ |
| 2 week | 59.52±11.79 | 219.64±18.9^a^ | 42.86±8.91^b^ | 132.14±17.47^a,b,c^ |
| 3 week | 55.36±20.23 | 236.19±62.52^a^ | 38.1±8.13^b^ | 142.86±34.5^a,b,c^ |
| 4 week | 51.79±13.36 | 265.24±49.4^a^ | 52.38±22.42^b^ | 183.33±42.49^a,b,c^ |
| 5 week | 65.71±4.94 | 300±46.15^a^ | 29.52±12.94^b^ | 183.33±32.27^a,b,c^ |
| 6 week | 51.67±13.51 | 253.33±111.07^a^ | 44.64±11.47^b^ | 178.57±37.3^a,b,c^ |

^a^P＜0.05 vs. the control group; ^b^P＜0.05 vs. the T2DM group; ^c^P＜0.05 vs. the VBI group.

**Supplementary Table 3** Comparison of Excretion (g/rat·day)

| Time Point | Control group | T2DM group | VBI group | T2DM+VBI group |
| --- | --- | --- | --- | --- |
| -1 week | 28.79±12.73 | 32.57±7.63 | 23.86±3.98 | 27.86±9.41 |
| 0 week | 33.14±12.35 | 171.07±41.69^a^ | 27.57±14.06^b^ | 168.86±26.95^a,c^ |
| 1 week | 18.39±2.25 | 175.05±47.23^a^ | 13.14±3.76^b^ | 67.36±16.8^a,b,c^ |
| 2 week | 23.11±13.58 | 203.33±30.44^a^ | 21.79±9.43^b^ | 105.54±19.6^a,b,c^ |
| 3 week | 17.54±6.5 | 176±51.72^a^ | 25.07±15.39^b^ | 145.75±32.92^a,c^ |
| 4 week | 23.67±8.66 | 188.48±53.69^a^ | 19.67±4.79^b^ | 129.13±28.74^a,b,c^ |
| 5 week | 26.24±7.31 | 180.52±44.41^a^ | 20.62±9.73^b^ | 132.81±23.22^a,b,c^ |
| 6 week | 34±13.07 | 197.05±31.28^a^ | 30.89±12.62^b^ | 177.1±25.36^a,c^ |

^a^P＜0.05 vs. the control group; ^b^P＜0.05 vs. the T2DM group; ^c^P＜0.05 vs. the VBI group.

**Supplementary Table 4** Comparison of Body Weight (g/rat)

| Time Point | Control group | T2DM group | VBI group | T2DM+VBI group |
| --- | --- | --- | --- | --- |
| -1 week | 274.4±8.5 | 272.8±6.14 | 264.8±3.11 | 274±13.29 |
| 0 week | 379.6±15.09 | 350.6±6.66 | 356±10.05 | 362.8±11.17 |
| 1 week | 410.8±16.9 | 372.6±12.14^a^ | 363±9.97^a^ | 376.8±23.59 |
| 2 week | 438.8±19.04 | 406±16.32 | 405.8±11.21 | 398.8±22.6^a^ |
| 3 week | 475.8±29.42 | 425.2±20.18^a^ | 441.8±16.24 | 419.6±33.92^a^ |
| 4 week | 503±21.35 | 449.4±26.76^a^ | 483.6±19.26 | 447.2±21.29^a,c^ |
| 5 week | 534.2±33.94 | 450±34.58^a^ | 513.8±21.45^b^ | 466.2±25.37^a,c^ |
| 6 week | 557.8±39.33 | 441.8±23.84^a^ | 553.4±22.41^b^ | 477.4±15.92^a.b,c^ |

^a^P＜0.05 vs. the control group; ^b^P＜0.05 vs. the T2DM group; ^c^P＜0.05 vs. the VBI group.

**Supplementary Table 5** Comparison of Blood Glucose (mmol/L)

| Time Point | Control group | T2DM group | VBI group | T2DM+VBI group |
| --- | --- | --- | --- | --- |
| -1 week | 9.04±0.57 | 8.44±1.05 | 7.96±0.84 | 8.04±1.18 |
| 0 week | 8.48±1.62 | 27.56±2.25^a^ | 8.12±1.48^b^ | 24.04±3.03^a,c^ |
| 1 week | 8.96±1.15 | 28.82±3.01^a^ | 8.1±0.91^b^ | 15.62±3.66^a,b,c^ |
| 2 week | 7.26±1.39 | 28.94±1.8^a^ | 7.98±0.88^b^ | 21.28±4.67^a,b,c^ |
| 3 week | 8.4±0.49 | 27±2.34^a^ | 6.7±1.29^b^ | 26.54±1.88^a,c^ |
| 4 week | 9.08±1.48 | 29.46±2.92^a^ | 8.24±0.93^b^ | 23.46±4.26^a,b,c^ |
| 5 week | 8.86±0.63 | 29.28±2.58^a^ | 7.62±0.87^b^ | 26.18±3.57^a,c^ |
| 6 week | 7.76±0.98 | 29.06±2.55^a^ | 8.34±0.86^b^ | 23.54±4.27^a,b,c^ |

^a^P＜0.05 vs. the control group; ^b^P＜0.05 vs. the T2DM group; ^c^P＜0.05 vs. the VBI group.
